# Influence of handwriting skills during spelling in primary and lower secondary grades

**DOI:** 10.3389/fpsyg.2013.00818

**Published:** 2013-11-05

**Authors:** Virginie Pontart, Christel Bidet-Ildei, Eric Lambert, Pauline Morisset, Lisa Flouret, Denis Alamargot

**Affiliations:** ^1^CHArt Laboratory, University of Paris 8Paris, France; ^2^CeRCA Laboratory, Centre National de la Recherche Scientifique-UMR 7295 University of PoitiersPoitiers, France; ^3^ESPE de l'Académie de Créteil, University of East Paris - CréteilParis, France

**Keywords:** handwriting, spelling, graphomotor skills, orthographic knowledge, temporal measures

## Abstract

We sought to identify, the impact of handwriting skills on the efficiency and temporal course of word spelling across Grades 2–9. Eighty-four students, drawn from primary and lower secondary schools, were asked to perform a dictation task to assess their word spelling. They also had to write out the letters of the alphabet, as well as their firstnames and surnames, from memory to assess their handwriting skills. Handwriting kinematics were recorded using a digitizing tablet and a computer running Eye and Pen software. Results revealed that graphomotor skills (as assessed by the name writing task) influenced the success and temporal course of spelling, but only in primary grades, whereas the influence of orthographic knowledge (as assessed by the alphabet task) could still be observed in the lower secondary grades, even if it ceased to influence the temporal course and only affected errors. We discuss what these findings tell us about changes in transcription processes over the course of child development.

## Theoretical framework

### Introduction

According to Berninger and Swanson (1994), the development of written production between Grades 1 and 9 can be divided into three stages: (i) the gradual emergence, during the lower primary grades, of the three main writing components (formulation, then revision, and finally planning); (ii) the complexification, during the upper primary grades, of their constituent processes and operations, allowing increasingly large units (letters, words, sentences, then paragraphs) to be taken into account; and (iii) the emergence, during the lower secondary of the ability to undertake parallel and interactive processing (between and within the three components). This development is highly constrained by working memory (WM) capacity. Owing to limited cognitive resources, processes have to compete with each other whenever overall demand exceeds supply. According to capacity theory, the gradual automatization of lower-level processes, such as handwriting, frees up resources that can then be used to engage more controlled (i.e., higher-level like spelling process) processes in parallel and/or to conduct more complex and demanding processing (Just and Carpenter, 1992; McCutchen, 1996, 2011).

The development of handwriting takes time and practice. For instance, the motor programs involved in handwriting do not emerge until around 8 or 9 years (Hamstra-Bletz and Blot, 1990), and are only automatized at around 14 years (Ajuriaguerra et al., 1971; Chartrel and Vinter, 2004, 2006). Writers who have not yet mastered handwriting have to attend to the lower-level skills that tax WM resources, thus, interfering with higher-order processes, such as text composition and/or spelling.

### Influence of handwriting skills on text composition and spelling

There is a large body of evidence to show that handwriting skills affect both text composition (Graham et al., 1997; Jones and Christensen, 1999; Connelly et al., 2005, 2012; Medwell et al., 2007, 2009; Morin et al., 2012) and spelling (Berninger and Alsdorf, 1989; Berninger et al., 1991; Graham et al., 1997; Fayol and Miret, 2005; Medwell et al., 2007; Abbott et al., 2010; Bourdin et al., 2010, for discussion; Connelly et al., 2012; Morin et al., 2012; Puranik and AlOtaiba, 2012).

Composing a good-quality text (i.e., coherent, cohesive, and respecting linguistic and communicative norms) is a demanding activity, and requires the Planning, Formulation and Revision components to be coordinated in WM (Alamargot and Chanquoy, 2001). Researchers have shown that graphomotor automatization, as assessed by the 60-s alphabet writing task (i.e., writing out the letters of the alphabet in the right order as fast as possible and within 1 min; see Berninger et al., 1991) explains a substantial proportion of the variance in text quality. This relationship appears early on in childhood (in Grade 1, Graham et al., 1997; Jones and Christensen, 1999; Medwell et al., 2007; in Grade 2, Morin et al., 2012) and persists across development. At Grade 5, for instance, graphomotor automatization continues to account for 21.5% of the variance in text quality (Medwell et al., 2009) and 30.5% of the variance in the number of words written between two pauses (Connelly et al., 2012). Interestingly, this relationship is also observed in adults when time pressure is applied. Therefore, even in adults, when mental load is high, the level of graphomotor automatization becomes a predictor of composition quality by modifying the engagement of the three writing components (Connelly et al., 2005).

Spelling relies on two sets of processes, depending on word frequency (for a review, see Tainturier and Rapp, 2001; Bonin, 2003). For frequent words, the letter sequence is directly retrieved from the orthographic lexicon via the lexical route. For rare or unknown words, the letter sequence is arrived at indirectly, through a process of phoneme–grapheme conversion (assembled route). The resulting orthographic representation is stored in a graphemic buffer (Caramazza et al., 1987; Hillis and Caramazza, 1989) until its graphomotor execution (i.e., initiation and implementation of motor programs and neuromuscular execution). Although the orthographic representation obviously needs to be retrieved or calculated prior to execution (i.e., during the writing latency; Bonin et al., 2002), Delattre et al. (2006) have shown that the orthographic processing of irregular and infrequent words is still generally incomplete at this stage, and therefore, continues during the graphomotor execution, thus, increasing handwriting duration. According to the authors, these results can be interpreted within the dual-route model. When a conflict arises between the outputs of the lexical and assembled spelling routes, it takes time to resolve, and the central processing of this conflict then cascades onto the graphomotor execution.

The fact that the spelling process can take place during handwriting is coherent with van Galen's cascading model (1991), according to which the central processing of the next word (or next part of the word) is engaged during the execution of the orthographic form of the previous word (or previous part of the word) stored in the graphemic buffer. This flexibility in the coordination of spelling during handwriting has been confirmed and explored further by Lambert et al. (2011); Roux et al. (2013) in adults, and by Maggio et al. (2012) in children.

Three factors seem to determine the temporal course of the central processing of spelling. The first one concerns the word's lexical characteristics. The less frequent and/or regular the word, the longer the central processing takes and the more likely it is to be cascaded. The second factor concerns time pressure. Until now, the cascading phenomenon has only been observed for the production of successive words under time pressure (Delattre et al., 2006; Lambert et al., 2011; Maggio et al., 2012). Producing a single word without any time constraint should minimize cascading, by allowing the spelling process to be completed during the pause (i.e., latency) preceding execution. Third, according to capacity theory, the ability to cascade processing depends heavily on the writer's development and expertise (Berninger and Swanson, 1994; Alamargot et al., 2010). Because handwriting is not yet fully automatized in younger writers, especially those still in primary school (Bourdin and Fayol, 2000; Olive et al., 2009), they have to prevent overloading by segmenting and sequentializing the higher processes and are therefore, unable to engage in parallel, or cascaded, processing (see Olive and Kellogg, 2002; Alamargot et al., 2007).

While spelling during handwriting can modify the temporal course of graphomotor execution (handwriting obviously has to wait for spelling), several studies have shown that handwriting skills can, in return, influence spelling performances. This influence was first demonstrated by Berninger et al. (1991) among children at the end of kindergarten (see also DeBruyn et al., 1985). The authors showed that performances on the alphabet writing task were significantly correlated with spelling achievement at this stage- a finding subsequently confirmed by Puranik and AlOtaiba (2012). This relationship has also been demonstrated in older children (Grade 1, Medwell et al., 2007; Grade 2, Morin et al., 2012; Grade 5, Medwell et al., 2009; Connelly et al., 2012), using a variety of tasks, including dictation (scored according to Tangel and Blachman's criteria, 1992), picture naming and the spelling of words, presented either singly or in context. This effect has been replicated in French. Fayol and Miret (2005) found a relationship in Grade 3 between handwriting skills (i.e., the level of automatization, as assessed by the 15-s alphabet writing task; Abbott and Berninger, 1993) and the number of orthographic errors (spelling and grammar) made during the dictation of a short text. Some studies (e.g., Graham et al., 1997) have indicated that the relationship between spelling and handwriting skills tends to wane beyond the lower primary level (Grades 1–3). However, when Abbott et al. (2010) tracked two cohorts from Grades 1 to 5 and from Grades 3 to 7 in a longitudinal study, modeling different sets of relationships between handwriting, spelling, text composition and reading, they found a significant correlation between handwriting automatization (as assessed by the 15-s alphabet task) and spelling performance (as assessed by the word dictation task of the Wechsler Individual Achievement Test; WIAT II- Wechsler, 2005) between Grades 3 and 4–5.

### Exploring the influence of handwriting skills further

The above studies have shown that handwriting skills influence text quality, text fluency and spelling performance. This influence is particularly noticeable between kindergarten and Grade 3, but can also be observed in Grades 4–5 and even, to a limited extent, in undergraduates. Although the existence of this influence is therefore, no longer in dispute, three important questions remain unanswered.

- The first of these concerns the descriptions and explanations given so far for the influence of handwriting skills on spelling. Most studies cite capacity theory (Just and Carpenter, 1992; McCutchen, 1996, 2011), arguing that handwriting diverts precious attentional resources away from the controlled processes involved in spelling (Fayol and Miret, 2005). While this is a valuable explanation, it remains general and does not tell us exactly how handwriting affects spelling. Because these studies relied heavily on orthographic error analyses, they generally assumed that the consequence of competition for attentional resources is a failure in spelling's central processes. Nevertheless, according to Just and Carpenter (1992), a shortage of resources does not systematically lead to errors. It can also cause processes to slow down and make the storage of representations more difficult, leading to some processes having to be reiterated. It might, therefore, be worthwhile enhancing the current method for analyzing spelling errors by undertaking more fine-grained analyses based on kinematic measures obtained by recording the temporal course of handwriting. Variations in speed and duration would reflect the degree to which higher-level processes needed to be sequentialized and/or reiterated during execution (Chesnet and Alamargot, 2005; Alamargot et al., 2006). Furthermore, we can only acquire a deeper understanding of graphomotor influences on spelling during handwriting if we distinguish between its effects on processing (i.e., on cascaded central processing) and its effects on storage (i.e., on the stored orthographic form). When cascading is either not possible (in younger children) or not necessary (no time pressure), poor handwriting skills should only affect the storage of the orthographic form in the graphemic buffer. However, when cascading is either possible (in older children and adults) or necessary (time pressure), poor handwriting skills should hinder not just the storage of the orthographic form, but also its central processing.
- The second question concerns the decrease across grades in the influence of handwriting skills. Although their effect on text composition has been investigated up to undergraduate level, their effect on spelling does not appear to have been examined beyond Grades 6–7. And yet, given that the automatization of handwriting increase with age up to 14–15 years (e.g., Ajuriaguerra et al., 1971), it is reasonable to assume that these skills cease to influence spelling in the lower secondary grades. However, this has yet to be confirmed.
- The third and last question concerns the methods used to assess handwriting skills thus, far. In the wake of work by DeBruyn et al. (1985), Berninger and Alsdorf (1989) and Abbott and Berninger (1993), researchers wholeheartedly adopted the alphabet writing task, first within 60 s, then within 15 s. The advantage of this task is that it probes both fluency (number of letters produced within a limited space of time) and quality (only letters that are correctly formed, legible and produced in the right order are taken into account). Even so, its interpretation is not as clearcut as might first appear. Although the task obviously probes graphomotor skills, pupils are seldom called upon to perform the written recall of the alphabet in the classroom (teachers prefer to use alphabet rhymes or songs), and the serial writing of isolated letters can be quite constraining, especially in the cursive handwriting favored by the French school system. Systematically leaving gaps between the letters and segmenting graphemic units that are increasingly chunked across grades very probably requires executive operations (inhibition of the urge to join the letters up and insertion of spaces). Participants also have to write the letters in the right order. Nor do performances on this task reflect solely graphomotor aspects. By its very essence, the written recall of the alphabet also relies on orthographic knowledge about the names and sounds (i.e., basic phoneme–grapheme correspondences) of the letters. This aspect of orthographic knowledge plays a decisive role in the development of literacy and spelling skills (Caravolas et al., 2001; Ehri, 2005; for a review, see Foulin, 2005), and may partly account for the variance shared by alphabet writing performances and spelling performances. For all these reasons, it would be useful to improve the assessment of handwriting skills by using a more ecological task than alphabet writing-one that avoids units being segmented and does not rely as heavily on orthographic knowledge. With this in mind, Alamargot et al. (2007) asked adults to write their firstnames and surnames out several times on a digitizing tablet, in order to record their handwriting kinetics. The authors were able to demonstrate that the handwriting speed recorded in this task was an accurate reflection of graphomotor automatization and partly explained the fluency observed in a subsequent text composition task (see also Chuy et al., 2012). The name writing task is useful because it limits the involvement of orthographic knowledge, does not require letters to be written separately, and relies on phoneme–grapheme correspondences that have been memorized at an early age and can be directly retrieved. Furthermore, students frequently have occasion to write their names in the course of their school work. Name writing thus, involves the best known and doubtless most automatized grapheme string.

### Rationale

In order to enhance our understanding of how the influence of handwriting skills on spelling performances changes across grades, we (i) administered a dictation task to pupils in primary (Grades 2–5) and lower secondary school (Grades 6–9), to pinpoint changes in spelling performance, as assessed by the number of spelling errors and the temporal course of handwriting to dictation, and (ii) measured changes in the impact of handwriting skills on spelling performances across grades. As recommended by Fayol and Miret (2005), and because lexical characteristics can influence the temporal course, we opted for a single-word dictation task, as this allowed us to control the frequency and regularity of the items more carefully. In order to better control the temporal course of spelling, as well as the processes engaged during handwriting to dictation, we decided not to impose any time pressure. Participants were free to write at their usual speed and were not required to write the same word out several times, as they were in Delattre et al.'s (2006) study. Moreover, in order to maximize the central processing of spelling during the writing latency (and therefore, limit a cascading effect as far as possible in the lower secondary grades), the word was read out twice and participants were asked to wait for a signal before starting to write. This procedure was intended to separate the central processing from the storage of the orthographic form as much as possible, as we wished to focus here more on graphemic buffering than on cascaded processing.

To assess the participants' handwriting skills, in addition to performing the alphabet writing task (Abbott and Berninger, 1993), they were asked to write out their firstnames and surnames (Alamargot et al., 2007; Chuy et al., 2012). Furthermore, in order to assess the temporal course of spelling in greater depth, as well as the level of graphomotor automatization, we systematically recorded the kinematics of the pen by using a digitizing tablet. Two variables were assessed: pen movement speed (cm/s), which excluded handwriting pauses and only included the times when the pen was moving across the tablet surface (Variable 1) and handwriting duration (ms/car) which did include handwriting pauses (Variable 2). The first variable was used specifically to assess the level of motor program automatization and the efficiency of neuromuscular execution, whereas the second one was used to assess higher-level processes, such as orthographic processing, in addition to motor programs and neuromuscular execution.

We hypothesized that grade-related changes in *spelling performances* in the dictation taks would take the form of (i) a reduction in the number of spelling errors, (ii) an increase in pen movement speed, owing to the gradual automatization of motor programs (see Hamstra-Bletz and Blot, 1990; Chartrel and Vinter, 2006), and (iii) a decrease in handwriting duration, stemming not only from graphomotor automatization, but also from more efficient storage of the orthographic form.

Regarding *handwriting performances*, changes would take the form of (i) an increase in pen movement speed for both handwriting tasks (name writing and alphabet), and (ii) a decrease in handwriting duration, but with longer durations in the alphabet writing task than in the name writing one, especially in the primary grades, due to their less efficient orthographic knowledge (i.e., letter names and sounds).

As for the impact of handwriting skills on spelling performance, we predicted that:

- if pen movement speed does indeed reflect the level of automatization of the graphomotor programs and motor execution, we would observe a strong and systematic correlation between the three writing tasks, whatever the grade, confirming that all three tasks involve the same basic graphomotors skills;
- in the primary grades, the combination of a lack of graphomotor automatization and less efficient orthographic knowledge would place a heavy burden on attentional resources, making it difficult to maintain the orthographic form during its graphomotor execution. As a consequence, during the dictation task, spelling errors, and handwriting duration would increase when handwriting skills-as assessed by pen movement speed and handwriting duration in the name writing and alphabet tasks-were less efficient;
- in the lower secondary grades, assuming that the graphomotor programs were increasingly automatized, only less efficient orthographic knowledge would make it difficult to maintain the orthographic form during its execution. As a consequence, in the dictation task, spelling errors and handwriting duration would increase when handwriting duration increased during the alphabet task, but not when it increased during the name writing task.

## Materials and methods

### Participants

Participants were 84 s to ninth graders drawn from four public schools in the Poitou-Charentes region of France. French was their mother tongue and their main means of communication. None of them had learning difficulties that were known at the time of the investigations or were discovered subsequently. The participants were divided into two groups (44 primary graders and 40 lower secondary graders), each encompassing four grades: Twenty-Seven Percentage pupils aged 6.92–7.69 years in Grade 2 (*M* = 7.42, *SD* = 0.22), 36% pupils aged 7.78–9.96 years in Grade 3 (*M* = 8.55, *SD* = 0.53), 20% pupils aged 8.72–10.02 years in Grade 4 (*M* = 9.44, *SD* = 0.37), and 16% pupils aged 9.23–11.97 years in Grade 5 (*M* = 10.66, *SD* = 0.80) in the primary group; and 38% pupils aged 10.96–12.67 years in Grade 6 (*M* = 11.53, *SD* = 0.48), 23% pupils aged 10.68–13.47 years in Grade 7 (*M* = 12.53, *SD* = 0.83), 20% pupils aged 12.61–14.81 years in Grade 8 (*M* = 13.39, *SD* = 0.69), and 20% pupils aged 14.18–15.37 years in Grade 9 (*M* = 14.69, *SD* = 0.47) in the secondary group. The groups contained 57 and 63% girls and 20 and 13% left-handed students, respectively.

### Tasks

#### Handwriting tasks

In the name writing task, participants had to write their firstnames and surnames out three times in a row, in their usual handwriting. The alphabet writing task, inspired by Abbott and Berninger (1993), consisted in writing out all the letters of the alphabet in the right order in lower-case letters, again in the participants' usual handwriting. They were told not to separate the letters with commas. In each task, they were instructed to write as quickly as possible but also as carefully as possible, in their usual handwriting.

#### Spelling task

The spelling task took the form of a dictation of 27 common nouns containing between five and eight letters, and between two and three syllables. With the help of the NOVLEX database (Lambert and Chesnet, 2001), we constructed a list of words (see Appendix) that varied in frequency and exemplified three levels of regularity (no phoneme–grapheme irregularity, one phoneme–grapheme irregularity, two phoneme–grapheme irregularities). As explained in the Rationale (1.4), participants were told to wait until the word had been read out twice before starting to write it.

### Apparatus

All three writing tasks were performed on a digitizing tablet (Wacom Intuos 3; 200 Hz sampling rate) hooked up to a laptop computer (Apple MacBook, piloted by Eye and Pen software; Chesnet and Alamargot, 2005; Alamargot et al., 2006). The software (i) managed the display of the instructions and the presentation of the dictation items, and (ii) recorded the position and pressure of the pen on the tablet's surface, together with the relevant speeds and durations. The participants wrote with a magnetic pen (Wacom Ink Pen) on the lined pages of an exercise book placed on top of the tablet. The 27 repeated words used in the spelling task had previously been recorded on 27 separate audio files, all lasting exactly the same amount of time. Pupils wore headphones so that they could hear the words clearly without disturbing the other participants.

### Procedure

The participants were assessed in groups of two or four in a quiet room in their school. Each session lasted approximately 30 min. Participants worked by themselves on their own digitizing tablet and computer setup. The tasks were administered in the same order for all the participants: the two handwriting tasks (name writing, then alphabet writing) first, followed by the spelling task. The order of the 27 dictation items was also fixed, based on increasing orthographic (frequency × regularity) complexity. The procedure in the spelling task was as follows: after a waiting screen had appeared, the experimenter pressed the space bar to trigger the recording by the Eye and Pen software and the playing of the first audio file. When the word had been played twice, a light flashed on the screen to tell participants that they could start to write the word on the page in the exercise book. When the participants deemed they had finished writing the word, they pressed the pen tip on the “I've finished” space on the tablet. This pen pressure prompted the display of a fresh waiting screen.

### Measured variables

In order to assess spelling efficiency during the dictation task, we worked out the percentage of incorrect words (i.e., containing at least one error). In order to assess the temporal course of handwriting, for each individual word, name and alphabet series produced, we calculated (i) pen movement speed (cm/s), and (ii) handwriting duration per letter (ms/car), as defined in the Rationale (1.4).

## Results

### Analysis of spelling performances

In order to describe the effect of grade on spelling performances, we ran a Student's *t*-test for each of the variables, with the two grade groups as the between-participants factor. Regarding spelling efficiency, results (Table 1) showed that the *percentage of incorrect words* decreased significantly with grade, *t*_(82)_ = 6.09, *p* < 0.0001. Of all the participants, only two secondary pupils made no mistakes. Regarding the temporal course of handwriting, *pen movement speed* increased significantly with grade, *t*_(82)_ = −4.72, *p* < 0.0001, but there was no significant difference between the two groups for *handwriting duration, p* > 0.45.

**Table 1:** **Spelling performances in the primary and lower secondary grades: mean (standard deviation) percentage of incorrect words, pen movement speed (cm/s) and handwriting duration (ms/car)**.

	**Primary grades**	**Secondary grades**
Incorrect words (%)	33.01 (13.87)	17.78 (7.96)
Pen movement speed (cm/s)	2.04 (0.53)	2.71 (0.75)
Handwriting duration (ms/car)	1748 (677)	1665 (168)

### Analysis of handwriting skills and influence on spelling performance

#### Handwriting skills

In order to describe the effect of grade on task-related handwriting skills, we ran ANOVAs for each of the measures, with grade as a between-participants factor and writing task as a within-participants factor.

***Pen movement speed.*** Results (Table 2) showed a significant main effect of grade, *F*_(1, 82)_ = 62.24, *MSE* = 0.52, *p* < 0.0001, and task, *F*_(1, 82)_ = 16.58, *MSE* = 0.06, *p* < 0.0002. Moreover, we found a significant Grade × Task interaction, *F*_(1, 82)_ = 12.59, *MSE* = 0.06, *p* < 0.001.

**Table 2:** **Handwriting performances in primary and lower secondary grades: mean (standard deviation) pen movement speed (cm/s) and handwriting duration (ms/car) for each handwriting task (name writing and alphabet writing)**.

	**Primary grades**		**Secondary grades**	
	**Name**	**Alphabet**	**Name**	**Alphabet**
Pen movement speed (cm/s)	1.55 (0.48)	1.53 (0.45)	2.56 (0.62)	2.28 (0.59)
Handwriting duration (ms/car)	1657 (533)	3185 (961)	766 (220)	1440 (391)

Newmans-Keuls *post-hoc* comparisons showed that pen movement speed was lower for alphabet writing than it was for name writing in the lower secondary grades, *p* < 0.001, whereas this difference was not significant in the primary grades.

***Handwriting duration.*** There were significant main effects of grade, *F*_(1, 82)_ = 145.17, *MSE* = 501289.5, *p* < 0.0001, and task, *F*_(1, 82)_ = 222.82, *MSE* = 227679.6, *p* < 0.0001. The Grade × Task interaction was significant, *F*_(1, 82)_ = 33.58, *MSE* = 227679.6, *p* < 0.0001. Newmans-Keuls *post-hoc* comparisons showed that handwriting duration was significantly higher during alphabet writing than during name writing, in both the primary, *p* < 0.001, and lower secondary, *p* < 0.001, grades, although the interaction showed that this difference was smaller in the lower secondary grades.

#### Influence of handwriting skills on spelling

In order to understand the influence of handwriting skills on spelling efficiency and temporal course, and to make a distinction between graphomotor skills (as assessed by the name writing task) and orthographic knowledge (as assessed by the alphabet writing task), we calculated Pearson correlation coefficients between handwriting and spelling performance, for each grade group (Table 3).

**Table 3:** **Pearson correlation coefficients (r) and probability for primary and lower secondary groups between handwriting performances (pen movement speed (cm/s) and handwriting duration (ms/car), in the name writing and alphabet writing tasks) and spelling performances [% of incorrect words, pen movement speed (cm/s) and handwriting duration (ms/car)]**.

			**Handwriting performances**			
			**Pen movement speed (cm/s)**		**Handwriting duration (ms/car)**	
			**Name**	**Alphabet**	**Name**	**Alphabet**
Spelling performances	Primary grades	Pen movement speed (cm/s)	0.77^**^	0.75^**^	–	–
		Handwriting duration (ms/car)	–	–	0.80^**^	0.41^**^
		Incorrect words (%)	−0.34^*^	−0.44^**^	0.38^*^	0.46^**^
	Secondary grades	Pen movement speed (cm/s)	0.73^**^	0.72^**^	–	–
		Handwriting duration (ms/car)	–	–	0.26	−0.01
		Incorrect words (%)	0.01	−0.15	0.02	0.41^**^

* p < 0.05;

** p < 0.01; – no correlation was calculated.

Regarding *pen movement speed*, results showed significant and positive correlations between handwriting and spelling tasks, in both the primary grades (*r* = 0.77 for name writing; *r* = 0.75 for alphabet writing) and lower secondary grades (*r* = 0.73 for name writing; *r* = 0.72 for alphabet writing task). Regarding *handwriting duration*, significant positive correlations only appeared in the primary grades, between spelling and the name writing (*r* = 0.80) and alphabet writing (*r* = 0.41) tasks. Regarding *spelling efficiency*, in the primary grades, the percentage of incorrect words was significantly and negatively correlated with the two handwriting tasks for pen movement speed (*r* = −0.34 for name writing; *r* = −0.044 for alphabet writing) and for handwriting duration (*r* = 0.38 for name writing; *r* = 0.46 for alphabet writing). In the secondary grades, the percentage of incorrect words was only correlated with handwriting duration in alphabet writing (*r* = 0.41).

## Discussion

The objective of the present study was to identify the impact of handwriting skills on the efficiency and temporal course of word spelling across grades. It yielded three main results.

First, as expected, spelling performances improved across grades, as attested by the decrease in incorrect words and increase in pen movement speed. Nevertheless, contrary to our expectations, despite faster pen movements, it took the lower secondary graders the same amount of time to write from dictation as it did the primary graders. Following Delattre et al. (2006), we postulate that this relative slowdown in handwriting in the older and more skilled writers reflects the possibility of engaging in cascaded spelling, in accordance with capacity theory (Berninger and Swanson, 1994; Alamargot et al., 2010).

Second, as expected, and reflecting the automatization of graphomotor execution, handwriting performances increased across grades, as shown by changes in pen movement speed and handwriting duration in the name and alphabet writing tasks. The fact that handwriting duration was always longer for alphabet writing than for name writing, even in the lower secondary grades, confirms the presence of higher-level processes (orthographic knowledge) during the alphabet task. However, the finding that pen movement speed was slower for alphabet writing than for name writing in the secondary grades was unexpected. One plausible explanation for this relative slowdown in alphabet pen movement speed among the older students is that they had become capable of retrieving and setting the motor parameters for one letter while executing the previous one. This shift to cascaded alphabet production may therefore, have slowed down the pen movement speed. This interpretation is consistent with the results of the spelling task, indicating an ability to cascade spelling. There was no such slowdown in name writing, as the grapheme string was retrieved from memory as a single chunk, thus, obviating the need to process the individual letters.

Third, our results confirmed the influence of handwriting skills on the efficiency and temporal course of spelling. Moreover, this influence changed across grades, undergoing qualitative rather than quantitative changes. The strong and systematic correlations between pen movement speeds for all three writing tasks, whatever the grade, confirmed that these tasks demand the same basic graphomotor skills.

In the primary grades, the systematic correlations between handwriting performances (for both tasks and both temporal variables) and spelling performances (for incorrect words and handwriting duration) confirmed that (i) orthographic processing was completed during the execution of the dictated words, and (ii) was therefore, influenced by the graphomotor program and orthographic knowledge. In the primary grades, the lack of graphomotor automatization, combined with less efficient alphabet letter retrieval, seemed to place a heavy burden on attentional resources, (i) triggering errors by making it difficult to maintain the orthographic form during its execution, and (ii) increasing handwriting duration by forcing participants to halt or slow down their handwriting, in order to reiterate the central processing of spelling each time there was a storage failure.

In the lower secondary grades, the relationship between handwriting and spelling performances was restricted to a single correlation between handwriting duration in alphabet writing and the number of incorrect words in dictation. This result, coherent with the findings of Connelly et al. (2012) in fifth graders, is important for two reasons. First, the disappearance of any correlation with the name writing task suggests that the impact of handwriting skills in secondary grades no longer stems from the demands of graphomotor execution, but only from the efficiency of orthographic knowledge, as assessed in the alphabet task (letter names and sounds). Second, the lack of correlation between handwriting duration in alphabet writing and handwriting duration in dictation is rather surprising, even though handwriting duration in alphabet writing was correlated with spelling errors. One possible explanation is that the ability to cascade spelling, which the lower secondary graders seemed to possess, modifies time constraints. Processing upstream the portion of the word that will be executed downstream limits the direct impact of central processing on handwriting execution. Cascading therefore, ensures a constant output while the calculations are performed upstream. This explanation is particularly interesting because it also indicates why the lower secondary graders' handwriting duration was no different from that of the primary graders. This relative slowness probably absorbed hesitations related to the spelling calculation-or the reiteration of that calculation-when orthographic knowledge was less efficient. Consequently, our findings suggest that the influence of handwriting skills in lower secondary grades is related more to orthographic knowledge than to graphomotor execution, which is increasingly automatized.

Finally, our analysis of the influence of handwriting skills on spelling performances supported our general hypothesis of a grade-related change in the nature of that influence. In line with our predictions, the graphomotor skills assessed by the name writing task only influenced the efficiency and temporal course of spelling in the primary grades, while the influence of the orthographic knowledge assessed by the alphabet task persisted into the lower secondary grades, although it ceased to impact the handwriting temporal course and only affected errors. Our results support the capacity theory (Just and Carpenter, 1992; McCutchen, 1996, 2011) and are in line with findings demonstrating an improvement in graphomotor skills (Hamstra-Bletz and Blot, 1990; Chartrel and Vinter, 2004, 2006) with development. Interestingly, the shift in influence from graphomotor skills to orthographic knowledge coincided with the mastery of the motor production rules involved in letter writing, which occurs at around Grade 3 for the isochrony principle (Bidet-Ildei and Orliaguet, 2008), and around Grades 5–6 for the motor anticipation rule (Louis-Dam et al., 2000). Our experiment therefore, appears to show that mastering these rules (especially motor anticipation, as it makes parallel processing more accessible) is a prerequisite for the switch in influence in the lower secondary grades. However, further research is needed to confirm this hypothesis.

## Conclusion

The present study yielded in-depth knowledge about the influence of handwriting skills on spelling, in the wake of previous studies on the subject (Berninger and Alsdorf, 1989; Berninger et al., 1991; Graham et al., 1997; Fayol and Miret, 2005; Medwell et al., 2007; Abbott et al., 2010; Connelly et al., 2012; Morin et al., 2012; Puranik and AlOtaiba, 2012). It also shed light on two previously unanswered questions set out in the theoretical framework, and raised a number of new questions that will have to be addressed in further investigations.

Our main finding concerned the extent of the influence exerted by handwriting skills. We continued to find evidence of this influence in the lower secondary grades (Grades 6–9), but from more or less efficient orthographic knowledge and no longer from graphomotor skills. In order to understand age-related changes in the influence of handwriting skills more fully, it would be useful to (i) identify the point at which graphomotor execution ceases to exert an influence, by considering more than two groups of levels, but the succession of different grades with larger samples of students and (ii) extend our investigations to higher grades, in order to find out if and when orthographic knowledge also ceases to exert an influence.

Moreover, by performing online measures, we were able to show that the principles of capacity theory can be applied to changes in children's handwriting and spelling processes. We believe that the ability to engage in parallel orthographic and graphomotor processes, which seems to be present in lower secondary graders, plays a decisive role for, according to the cascade model (van Galen, 1991), it brings with it a new system of constraints and influences between lower- and higher-level processes, which henceforth have to interact. Although we now have a clearer understanding of how this system operates in adults (Lambert et al., 2011), future studies will have to clarify how it develops either in typically developing children or in children experiencing writing difficulties.

A Secondary finding concerned the temporal course of spelling during handwriting. The present study focused on handwriting and spelling in an experimental situation designed to minimize cascaded spelling, imposing latency and not introducing any time pressure. However, different indices show that even under these conditions, spelling is cascaded in the lower secondary grades. Further research in needed to confirm this presence by no longer controlling the latency period (e.g., using a picture naming task with free consultation) and by introducing time pressure (e.g., successive rapid copies). Experimental variations in the lexical (frequency) and sublexical (consistency) characteristics of the dictation items would help us to verify the presence of cascaded spelling, in the case of infrequent and/or irregular words (Delattre et al., 2006).

The last finding concerns the method used to assess the level of handwriting ability. Although alphabet writing is a useful test, it is not simply a measure of graphomotor automatization in handwriting, even if it is often used for that purpose by researchers. A more fine-grained method of measuring handwriting automatization-as well as a more suitable one in developmental terms-is to combine the alphabet writing task with a name writing one, as we did.

### Conflict of interest statement

The authors declare that the research was conducted in the absence of any commercial or financial relationships that could be construed as a potential conflict of interest.

## Appendix

Frequency, according to the NOVLEX lexical database, and regularity of dictation words.

**Table:**

**Irregularity 0**	**Irregularity 1**	**Irregularity 2**
Latin (238)	Chorale (238)	Hygiène (238)
Orbite (476)	Nylon (476)	Homard (476)
Piéton (714)	Sachet (714)	Boyau (714)
Donjon (1190)	Jumeau (1190)	Crapaud (2142)
Bijou (4284)	Gardien (5950)	Paysage (4284)
Sapin (6664)	Leçon (7378)	Fusil (7616)
Patron (9997)	Bureau (9521)	Haricot (8092)
Mouton (15709)	Hibou (10949)	Palais (24764)
Maman (118062)	Garçon (69742)	Sorcier (67362)
